# Screening recurrence and lymph node metastases in head and neck cancer: the role of computer tomography in follow-up

**DOI:** 10.1186/1758-3284-3-18

**Published:** 2011-03-25

**Authors:** Valentina Rivelli, Heinz T Luebbers, Franz E Weber, Claudia Cordella, Klaus W Grätz, Astrid L Kruse

**Affiliations:** 1 Department of Craniomaxillofacial and Oral Surgery, University Hospital Zurich, Zurich, Switzerland

## Abstract

**Introduction:**

Follow-up of patients with oral cancer is being questioned with regard to financial costs and effectiveness. Therefore, the aim of the present study was to evaluate whether local recurrence and cervical lymph node metastases were first discovered clinically or by routine computer tomography.

**Materials and methods:**

The records of all 317 patients that were treated for an oral cancer between 1998 and 2008 were systematically reviewed. Criteria for inclusion were tumor histology with a squamous cell carcinoma of the head and neck, and regular follow-up examinations with a minimum follow-up time of 12 months, including clinical and radiological (CT) controls. All patients had the first CT after 6 months, followed by yearly CT controls.

**Results:**

Out of 315 patients with an oral squamous cell carcinoma, 294 were evaluated. Those experiencing neither recurrence of the tumor nor lymph node metastases constituted 62%. Local recurrence was seen in 36 (12%), lymph node metastases in 32 (11%), and both in 16 (6%). Of the 32 patients with lymph node metastases, 25 were recognized first clinically, and 7 were detected by routine CT scans; concerning local recurrence, 32 appeared clinically, and 4 were detected by routine CT scans.

**Conclusion:**

Routine CT for follow-up is still indicated for detecting lymph node metastases as well as local recurrence.

## Introduction

The 5-year disease-specific survival rate for patients with primary oral cancer ranges from 53% [1] to 74% [2]. Most of the locoregional recurrences and lymph node metastases in head and neck cancer occur within the first two years [3,4]. The status of the lymph nodes appears to be one of the most important prognostic factors for patients with squamous cell carcinoma (SCC) of the head and neck, and recurrent disease seems to be associated with decreased survival [5]. Examination of the neck is based mainly on palpation.

Follow-up, including CT scans, of patients with oral cancer is being questioned with regard to financial costs and effectiveness. Most of the studies dealing with detection of cervical lymph node metastases are preoperative assessments. In surgically treated or radiated necks, the assessment seems to be more difficult due to scar tissue. Therefore, the aim of the present study was to evaluate whether local recurrence and cervical lymph node metastases were first discovered clinically or by routine computer tomography.

## Materials and methods

The records of all 317 patients treated for oral cancer between 1998 and 2008 at the Department of Craniomaxillofacial and Oral Surgery, University Hospital Zurich, were systematically reviewed. Criteria for inclusion were tumor histology with an SCC of the head and neck, and regular follow-up examinations, with a minimum follow-up time of 12 months, including clinical and radiological (CT) controls. Criteria for exclusion were inadequate information, tumors in other regions of the head and neck (e.g., salivary glands, skin), and patients with previous oral carcinoma. All patients had the first CT after 6 months, followed by yearly CT controls. Clinical controls were performed once per month in the first year; in the second year, every 2 months; in the third year, every 3 months; in the fourth year, every 6 months; and once per year after the fifth year (Table 1). Due to inadequate information, only 294 patients were included. Data of recurrence, lymph node metastases, and second head and neck cancer were analyzed.

**Table 1:** Follow-up schema

TIME	CLINICAL CONTROL	RADIOLOGICAL CONTROL
Year 1	monthly control	after 6 and 12 months

Year 2	every 2 months control	once a year

Year 3	every 3 months control	once a year

Year 4	every 6 months control	once a year

From year 5	once a year control	only in suspicious cases

## Results

**Figure 1:**
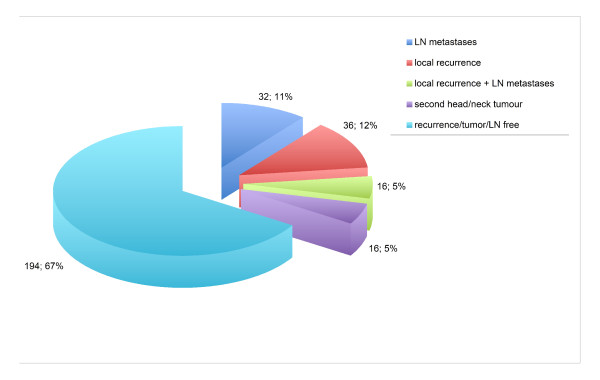
**Clinical and radiological assessment of recurrence and lymph node metastases**.

Out of 294 patients with an oral squamous cell carcinoma, the male-female ratio was 172:122 with a median age of 62.25 years; those experiencing no tumor recurrence or lymph node metastases constituted 62%. Local recurrence was seen in 36 (12%), lymph node metastases in 32 (11%), and both in 16 (6%) (Figure 1).

**Figure 2:**
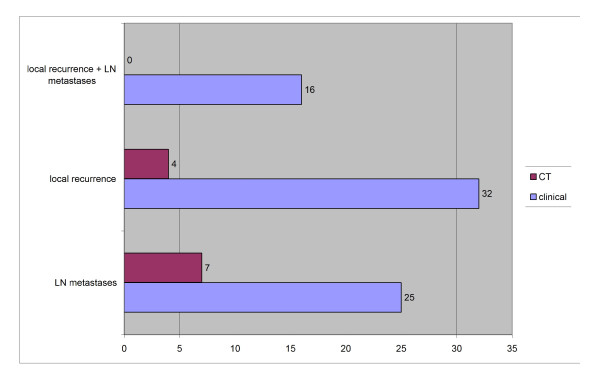
**Determination of follow-up results**.

Out of 32 patients with lymph node metastases, 25 were first recognized clinically, and 7 were detected by routine CT scans; concerning local recurrence, 32 appeared clinically, and 4 were detected by routine CT scans (Figure 2).

**Figure 3:**
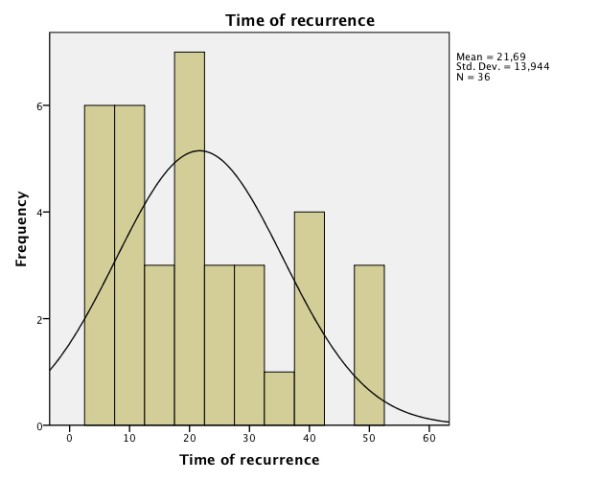
**Frequency and time of local recurrence (months)**.

**Figure 4:**
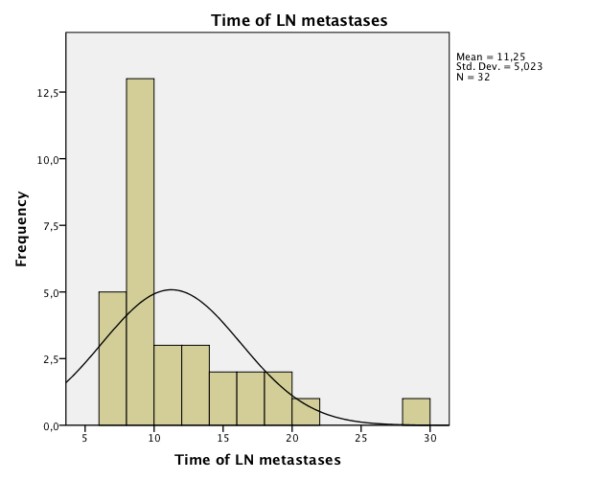
**Frequency and time of lymph node metastases (months)**.

**Figure 5:**
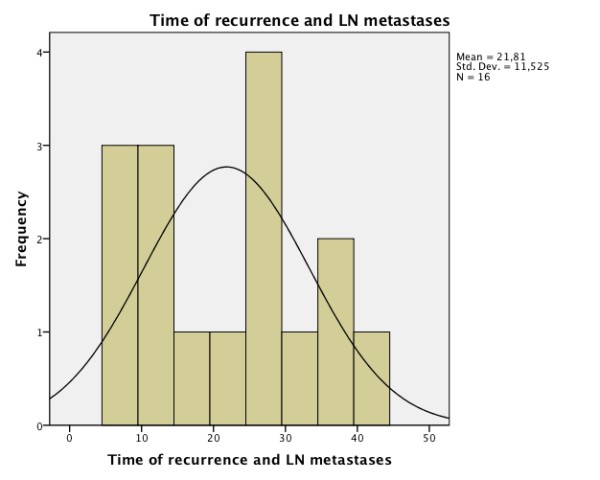
**Frequency and time of combined lymph node metastases and local recurrence (months)**.

Local recurrences (Figure 3) appear later than lymph node metastases (Figure 4). The combination of both (Figure 5) had the longest time to event. The exact data is given in Table 2.

**Table 2:** Data of recurrence and LN metastasis (Missing = Patients without local recurrence or LN metastasis during follow-up)

		Time of recurrence	Time of LN metastases	Time of recurrence and LN metastases
N	Valid	36	32	16
	
	Missing	48	52	68

Median		18,00	9,00	23,50

Std. Deviation		13,944	5,023	11,525

Minimum		5	7	7

Maximum		51	28	42

## Discussion

Palpation alone for assessment of cervical lymph node metastases seems to be unreliable [6]. For staging, ultrasonography, computed tomography, and magnetic resonance imaging (MRI) are generally considered superior to palpation [7]. Concerning the comparison between palpation, CT, and low field MRI, Atula et al. were able to show, in 86 patients without palpable normal necks, that CT (23 positive) was superior to low field MRI (10 positive) and ultrasonography (12 positive) [6]. Yousem et al. arrived at similar results by studying central nodal necrosis and extracapsular spread, experiencing a more accurate detection by CT in comparison to unenhanced or enhanced MRI [8]. In one of the largest meta-analyses, de Bondt et al. showed that ultrasonography-guided fine needle aspiration cytology had the highest diagnostic odds ratio (DOR = 260), compared to ultrasonography (DOR = 40), CT (DOR = 14), and MRI (DOR = 7) [9].

Nowadays, FDG-PET seems to play a more and more intensive role in lymph node metastasis or second tumor assessment. Recently Yamazaki et al. studied 1076 lymph nodes with preoperative FDG-PET and CT. FDG-PET detected 100% of metastatic lymph nodes ≥ 10 mm and intranodal tumor deposits ≥ 9 mm, and had fewer false-positives than did CT [10].

Several CT criteria for assessing nodal metastases have been discussed, like nodal size criteria (greatest diameter more than 1.5 cm for jugolodigastric and submandibular nodes, more than 1 cm for all other lymph nodes) nodal shape (more spherical shape in metastastic nodes), nodal grouping (three or more, each with a diameter of 8-15 mm), and central necrosis [11]. But in postoperative or radiated necks, the evaluation appears to be more difficult that in the preoperative status.

**Figure 6:**
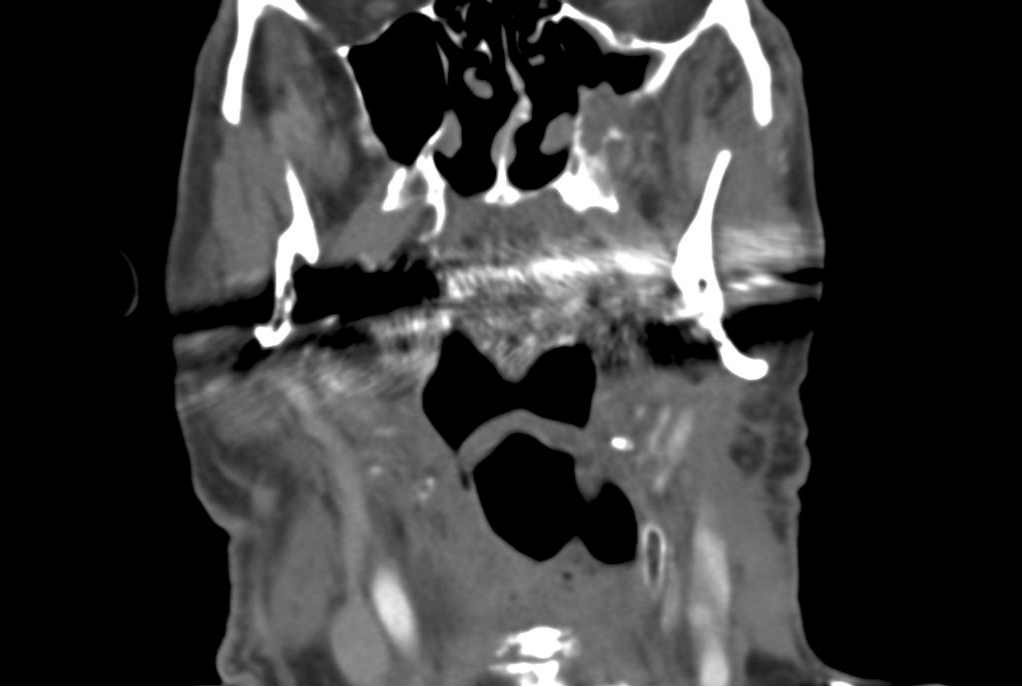
**Lymph node metastasis Level II detected by CT-scans**.

Some authors advocate the use of ultrasound due to good results in lymph node control [12] and lower costs, while others [8,13] prefer CT scans due to a higher sensitivity from CT imaging in comparison to ultrasound. One reason may be the better detection of deep cervical nodes by CT (Figure 6).

More than two-thirds of locoregional recurrences and lymph node metastases occur within the first two years [14,15]. In the present study, local recurrence (Figure 3) appeared later in comparison to lymph node metastases (Figure 4).

**Figure 7:**
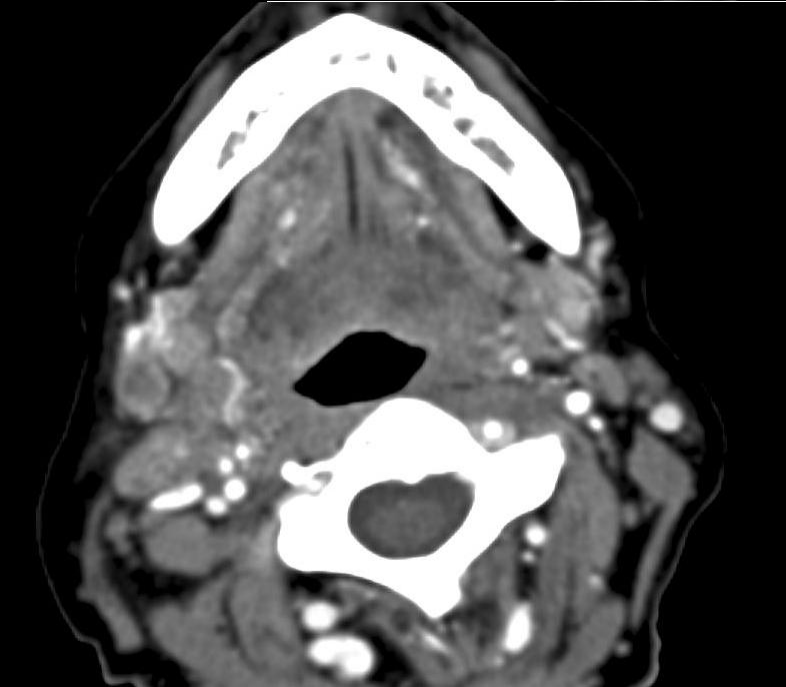
**Local recurrence of the maxilla first detected by CT-scans**.

Concerning detection of local recurrence by CT scans, data from the literature are not available. In the present study, local recurrence was detected first by CT scans in 4 patients (Figure 2); 3 out of these 4 were localized in the orbit and one in the maxilla (Figure 7). One reason could be that locations like mouth floor or tongue can be better observed. Therefore, in cases of poorer visual assessment, like in the reconstructed maxilla, CT can be advantageous for local control, whereas ultrasonography does not have a field of indication. Another alternative could be 18F-FDG PET/CT, but it is not available in all hospitals and is more cost intensive. Abgral et al., in 91 patients without clinical evidence of recurrence of head and neck SCC that were examined by 18F-FDG PET/CT, demonstrated proven recurrence in 30 patients [16].

The present study demonstrates that a reduction in the follow-up period of 5 years is not acceptable, in particular with regard to local recurrences. CT is still indicated for follow-up controls besides the clinical controls, but the alternative of ultrasonography, in particular for neck evaluation, should be taken into further consideration.

## Conclusion

Routine CT for follow-up is still indicated for detecting lymph node metastases as well as local recurrence. Ultrasonography does have a growing importance for detection of lymph node metastasis but not for local recurrences. Thorough clinical investigation is of course the baseline diagnostic.

## Competing interests

The authors declare that they have no competing interests.

## Authors' contributions

VR and CC carried out the retrospective study, HT and FW drafted the manuscript, MB participated in the design of the study, KW and AK participated in the design and coordination of the study. All authors read and approved the final manuscript.
